# Development and Validation of ICT Self-Efficacy Scale: Exploring the Relationship with Cyberbullying and Victimization

**DOI:** 10.3390/ijerph15122867

**Published:** 2018-12-14

**Authors:** Sadia Musharraf, Sheri Bauman, Muhammad Anis-ul-Haque, Jamil Ahmad Malik

**Affiliations:** 1 National Institute of Psychology, Quaid-i-Azam University, Islamabad 45320, Pakistan; haqanis@yahoo.com (M.A.); ja.malik@nip.edu.pk (J.A.M.); 2 Department of Applied Psychology, The Women University, Multan 66000, Pakistan; 3 Disability and Psychoeducational Studies, University of Arizona, Tucson, AZ 85721, USA; sherib@email.arizona.edu

**Keywords:** ICT self-efficacy, cyberbullying, cyber victimization, university students, Pakistan

## Abstract

The purpose of this study was to develop and validate the ICT Self-Efficacy Scale and the association of cyberbullying and victimization with ICT self-efficacy. Sample 1 (436 university students) was used to identify the factor structure of the Scale, and sample 2 (1115 university students) provided the data to confirm the factor structure (CFA), and to compute the internal consistency reliability, and convergent validity of the scale. Findings demonstrate that the new scale is a reliable and valid domain-specific measure to assess ICT Self-Efficacy for university students. Suggestions for further research with the scale are provided.

## 1. Introduction

The proliferation of Information and Communication Technology (ICT) and its utilization in higher education has brought drastic changes to various aspects of the learning environment, while facilitating easy online communication with peers. The changing trends in the use of World Wide Web following the delivery of web 2.0 technologies (websites that are interactive and include user-generated material) have revolutionized the ways in which ICT is used for learning and communication purposes [1].

Examples of web 2.0 sites that are increasingly used by students in higher education include social media (e.g., Facebook), wikis, blogs, video sharing sites (e.g., YouTube) folksonomies (“tagging” keywords on websites and links), etc. [2]. Although these advances in online technologies have proffered numerous benefits to students, they can also be used for nefarious purposes, one of which is cyberbullying [3,4].

Cyberbullying is defined as “any behavior performed through electronic or digital media by individuals or groups that repeatedly communicates hostile or aggressive messages intended to inflict harm or discomfort on others” [5] that involves a power imbalance between a victim and the perpetrator [6,7]. Typically, cyberbullying behaviors include sending intimidating or derogatory messages, spreading rumors, posting unwanted images or videos, revenge porn, posting offensive comments about someone, creating fake accounts using someone’s identity, and excluding someone out of an online communication with a malicious intent to hurt someone.

Researchers have described several differences between traditional and cyberbullying [8,9]. First, perpetrators can conceal or manipulate their identity during digital interactions. Second, perpetrators cannot view the immediate emotional effects on victims that consequently reduce guilt feelings and increase moral disengagement. Third, no geographical constraints exist in the online world and perpetrators can access the victims anytime-anywhere. Fourth, digital content (cyberbullying post) can often go viral with widespread distribution to a global audience, thus leading to greater humiliation. Fifth is the real or perceived power imbalance. For example, in traditional bullying, power might drive from physical strength, popularity, higher social status or older age [10] but in cyberbullying it might indicate superior technological expertise [11]. The act of cyberbullying (e.g., sending nasty text messages, posting compromising photos of others online, creating fake social network accounts, or posting someone’s videos online to damage his/her reputation) often requires very basic skills. However, there are more complex forms of cyberbullying, such as modifying pictures or hacking into someone’s social media account, that require more advanced skills [11]. 

Barlett and colleagues [12] reported that although cyberbullying perpetration was positively correlated with self-reported harmful online power and a positive attitude about cyberbullying, online power was not found to be a strong predictor of cyberbullying attitude and behavior. Rather, research on children and adolescent samples suggest that a higher probability for engaging in cyberbullying was associated with greater online expertise [13], computer skills [14], greater ICT expertise [15], risky usage of ICT [16], more time spent online, more frequent involvement in instant messaging [17], and heavier use of social networking sites [18].

The construct of self-efficacy was devised by Bandura and extensively used in educational, clinical, organizational, social, and health contexts [19]. Self-efficacy refers to individuals’ belief in their ability to accomplish a task [20]. The strength of perceived self-efficacy not only has a direct impact on the choice of activities and settings but it can also influence the ability to initiate coping efforts. Hence, self-efficacy changes across situations and circumstances instead of a general disposition [21].

This theory has provided insights to investigate the role of self-efficacy in bullying behavior. Self-efficacy has been addressed in both traditional and cyberbullying [22,23]. Previous research reported inconsistent findings concerning the relationship of perpetration of bullying and self-efficacy. Natvig and colleagues [24] reported that perpetration of bullying and general self-efficacy are significantly positively associated among adolescents, while others found a negative correlation [25]. Research has also been conducted to examine the relationship of cyberbullying with self-efficacy. For example, Olenik-Shemesh and Heiman [26] found that cyber victims reported lower levels of social and emotional self-efficacy than nonvictims. In other studies, general self-efficacy was found to be negatively associated with both perpetration of cyberbullying and cyber victimization [22,23]. As self-efficacy is a domain-specific construct, research has shown that adolescents’ high level of self-efficacy in their competence to engage in cyberbullying was positively associated with cyberbullying, and these beliefs were also found to be a moderator for the relationship of moral disengagement and cyberbullying [27].

Given the lack of attention paid to examining the relationship of cyberbullying with ICT self-efficacy, we found only one study that investigated how personal factors including social skills, trait verbal aggression, and Internet self-efficacy contribute to the perpetration of cyberbullying [28]. Findings revealed that Internet self-efficacy was not associated with the frequency of the perpetration of cyberbullying in college students. Rather, trait verbal aggression moderated the relationship between social skills and the frequency of the perpetration of cyberbullying only for those who had high Internet self-efficacy; this relationship was not found at the low level of Internet self-efficacy. However, the relationship of Internet self-efficacy with cyber victimization was not explored in this research. Further, cyberbullying was measured by a single item, and the validity and reliability of single-item measurement concerning cyberbullying has been considered problematic [29]. Moreover, the scale used to measure Internet self-efficacy [30], consists of items relating to Internet hardware, software, and the troubleshooting of Internet problems. This may not reflect the skills used in cyberbullying, particularly skills contributing to the perception of cyber superiority.

Similarly, Torkzadeh and Van Dyke [31] developed a multidimensional measure of Internet self-efficacy by focusing on general activities to interact with the Internet such as “surfing/browsing,” “encryption/decryption,” and “system manipulation,” rather than any specific organized activities. In a scale by Tsai and Tsai [32], items assess only knowledge and handling of web browser and searching skills. Thus, many of the advanced digital skills to measure ICT self-efficacy are not well captured by these earlier scales.

In addition, social media and other mechanisms through which cyberbullying and victimization occur have also evolved over time [33]. For example, in early studies on students, victims and perpetrators both reported instant messaging as the most frequently used venue for cyberbullying [34], while in another study, chatrooms were implicated in cyberbullying among adolescents [35]. However, Whittaker and Kowalski [36] found texting, Twitter, Facebook, Instagram, and YouTube as the most frequent venues, while instant messaging and chat rooms were infrequent places for cyberbullying and victimization. Such changes render older scales less relevant to the current context.

The majority of students today possess unprecedented digital skills, and ICT self-efficacy is not simply learning terms related to hardware and software or having searching skills. ICT self-efficacy might be a broader construct consisting of Internet usage behaviors that contribute to individuals’ perceptions of their control in cyberspace. Thus, efforts have been made to develop new measures that are consistent with the evolving and rapidly changing features of the technology. For instance, the Kim and Glassman [37] measure of Internet self-efficacy incorporated more socially complex skills and social experience on the Internet. Similarly, Chuang and colleagues [38] focused not only on usage, searching, and communication aspects but also included the Internet-related metacognitive skills and applications to measure ICT self-efficacy. Although these scales have incorporated sophisticated and advanced features of technology, they address the context of learning rather than interpersonal interaction.

The purpose of the present research is to develop and validate a domain-specific measure of ICT self-efficacy tailored to assess the relationship of cyberbullying and victimization with ICT self-efficacy in the context of interpersonal communication. Items were constructed considering venues like social networking sites where cyberbullying and victimization mostly take place, and included skills that perpetrators use to bully others and skills that can protect one from being victimized. The second objective of the study is to examine the relationship of cyberbullying and victimization with ICT self-efficacy. The research was conducted in two phases. Study 1 determined the factor structure of a newly developed ICT Self-Efficacy Scale, while study 2 analyzed the factorial and convergent validity of the scale and the relationship of cyberbullying and victimization with ICT self-efficacy.

## 2. Study 1: Development of ICT Self-Efficacy Scale and Exploratory Phase

Following Bandura’s [19] conceptualization, ICT Self-Efficacy is defined as one’s judgment of one’s ability to carry out activities required to successfully complete essential Internet and communication technology tasks, including skills related to social networking service (SNS) use. This definition was used to guide the development of the ICT Self-Efficacy Scale. Along with skills covered in existing measures, our intent was to include the “social networking service use” part of the definition that may be assessed by individuals’ behaviors on social networking sites. For item generation, existing studies were examined [37,38,39]. Items concerning skills for SNS usage were guided by information about safety and security skills available on popular SNS.

Bandura [20] described the magnitude, strength, and generality as three facets of self-efficacy. Magnitude focuses on the level at which one believes that he or she can complete the task, strength indicates how much belief one has to accomplish a particular task, and generality reflects the degree to which self-efficacy in one situation extends to another situation. In Bandura’s view [21], measurement of perceived self-efficacy entails the assessment of these three facets. Generally, researchers are more interested in specific rather than general self-efficacy; therefore, the dimension of generality is mostly excluded from the measurement of self-efficacy.

Bandura [19] suggested that the strength dimension is most important and therefore is essential to measure. Traditional measures of self-efficacy incorporated the dimensions of magnitude and strength [40,41], requiring respondents to respond to two items. To assess magnitude, participants are asked whether or not they can execute a certain level of specific task (yes/no), while, to assess strength, they are asked to indicate the percentage of confidence that they possess to perform a specific skill. These two responses are then combined to yield a composite self-efficacy score.

Researchers argued that a Likert scale measure of self-efficacy is more convenient because it only requires one response and is equivalent to the two-question format used in the traditional approach. A response indicating “Agree” is equivalent to “Yes” and a response indicating “Disagree” is equivalent to “No,” and the strength dimension can be measured by the distance away from the neutral response [42]. Further, research showed that traditional and Likert format scales of self-efficacy have similar psychometric properties: similar factor structures, equal levels of prediction and discriminability, and similar reliability–error variance. Thus, as proposed by researchers [41,43], a Likert scale format was used for the development of ICT self-efficacy scale in the present study.

Moreover, for ease of scoring and interpretation, item responses were on a 5-point Likert scale where 1 = Disagree Strongly and 5 = Agree Strongly. The responses were then treated on an interval scale in subsequent analyses. Experts have suggested that Likert scales can be analyzed effectively as interval scale when there are as many as four categories per variable [44,45]. The scale was developed in English as it is an official language in Pakistan. Although Urdu is the national language of Pakistan, English is taught at school in Pakistan, and the medium of instruction is English at the university level [46].

### 2.1. Content Validity

To ensure content validity, six expert panelists with prior experience conducting research on self-efficacy provided ratings of the suitability and appropriateness of an initial pool of 26 items to measure the desired construct. They were given definitions of self-efficacy and ICT self-efficacy and asked to rate the extent to which each item adequately represents the ICT self-efficacy construct definition. A 4-point scale ranging from (1) “Not Relevant” to (4) “Highly Relevant” was chosen, following Lynn [47], Waltz and Bausell [48]. To estimate the item-level content validity index (I-CVIs) for each item, the number of panelists who rated the item as either (3) or (4) was divided by the total number of panelists. Out of 26 items, five items were found to have I-CVIs less than 0.83 [47] and were thus removed from the pool. Three further items were revised by incorporating panelists’ suggestions for clarity of item wording. Further, a scale-level index (S-CVI) was computed on the final set of 21 items by calculating the average of I-CVIs across all 21 items. The S-CVI for a set of 21 items was found to be 0.87, which was above the recommended criteria of 0.80 [47].

### 2.2. Exploratory Factor Structure (EFA)

The factor structure was explored on the 21-item pilot scale using Principal Component Analysis (PCA).

## 3. Materials and Methods

### 3.1. Sample

A convenience sample of 436 Pakistani university students (120 males and 315 females; one respondent did not report gender, between the ages of 18 and 25 (*M* = 20.54, *SD* = 1.84) was used in the exploratory analysis. Participants were from six universities in Islamabad and Rawalpindi, Pakistan, and were enrolled either in bachelor’s or in master’s programs in social or natural sciences.

### 3.2. Measure

The initial ICT Self-Efficacy Scale was a self-report measure with 21 items. It scores on the 5-point scale ranging from “agree strongly” (5) through “uncertain” (3) to disagree strongly (1). Higher scores indicate greater ICT self-efficacy.

### 3.3. Procedure

Before survey administration, the study received approval from the ethical review board of the researcher’s university. The proposal of the study was evaluated by the board using the American Psychological Association ethical guidelines. The survey was anonymous and administered by the researcher during class hours. Demographic information was also collected. Respondents read and signed the voluntary consent form before participating in the survey.

## 4. Results

PCA was carried out to identify interpretable patterns in the ICT Self-Efficacy Scale. For the dataset, the value of Kaiser–Meyer–Olkin = 0.95 indicated an excellent measure of sampling adequacy. Bartlett’s Test of Sphericity (*χ*^2^ = 5348.61 (210), *p* < 0.001) was significant and provided additional evidence of the factorability of the data [49]. Following Guttman’s rule and examination of the scree plot, three meaningful factors with eigenvalues >1 were selected. Experts agree that the extraction of factors based on eigenvalues >1 often overestimates or underestimates the relevant components [50,51]. Similarly, the reliability of the scree plot is also questionable [52]. O’Connor [53] recommended more robust statistical procedures to determine the number of factors with higher precision—specifically, parallel analysis and Velicer’s minimum average partial (MAP) test. Parallel analysis and MAP, in combination, can lead to the most optimal decision because both tend to err in opposite directions, the former in the direction of overextraction and the latter in the direction of underextraction [53].

Accordingly, a parallel analysis [53] was performed in SPSS Version 22.0 (IBM Corp., Armonk, NY, USA). Data were normally distributed for all 21 items and the 1000 parallel data sets were used to run permutations of the raw data. Eigenvalues derived from the actual data were compared with eigenvalues derived from the randomly generated data. Results of the analysis yielded three significant values from the actual data and thus three factors were retained. These values were larger than the 95th percentile eigenvalues and the mean random data eigenvalues. Similarly, the MAP test was run in SPSS using a syntax by O’Connor [53]. Results obtained were in line with the parallel analysis and provided support for the retention of three components.

A second PCA was carried out with the direct oblimin rotation method, with extraction setting restricted to three components and item loading ≥ 0.40. These three components (21 items) explained a total of 61.23% of item variance. Results showed that 18 items appeared to have clear loading on their respective components. To avoid confusion, the three items with cross-loadings were excluded in further analyses.

Component one consisted of ten items with loadings ranging from 0.54 to 0.87 that explained 46% of items’ variance. Content analysis of the items showed that the items addressed issues relating to security and privacy. Thus, this component was labeled as “Privacy and Security.” Component two was comprised of five items with loadings ranging from 0.64 to 0.87, explaining an additional 8.38% of items’ variance. The items appeared to address individuals’ learning and the ability to judge and evaluate the content and users. The component was hence labeled “Differentiation and Learning.” Finally, the third component contained three items with loadings from 0.55 to 0.84 and explained 6.84% additional variance. Content analysis of the items indicated that these items assessed the individual’s ability of verbal and visual communication.

Thus, study one found that the ICT Self-Efficacy Scale consisted of three conceptually and statistically validated components. Alpha coefficients for the scale are provided in Table 1. 

## 5. Study 2: Confirmatory Phase

Study 2 was conducted to validate the factor structure of the ICT Self-Efficacy Scale using confirmatory factor analysis (CFA), and to access convergent validity on a new independent sample. 

## 6. Materials and Methods

### 6.1. Sample

The sample was composed of 1115 Pakistani university students (416 males, 699 females) between the ages of 18 and 25 years (*M* = 20.79, *SD* = 1.94) from six Pakistani universities in Punjab Province. A similar survey administration procedure was used as in study 1.

### 6.2. Measures

Along with the final 18-item ICT Self-Efficacy Scale, respondents completed the following measures which were then used to access validity:

#### 6.2.1. General Self-Efficacy Scale (GSES)

GSES is a 10-item measure to assess general perceived self-efficacy [54]. Items are rated on a 4-point scale from “not at all true” (1) to “exactly true” (4), yielding scores from 10 to 40, with higher scores indicating higher general self-efficacy. Sample items include, “I am confident that I could deal efficiently with unexpected events.” Cronbach’s alpha reliability for the GSES was 0.88 for the present study.

#### 6.2.2. ICT Use Scale

The original version of the ICT Use Scale was developed to examine adolescents’ use of ICT in a Swiss study [55]. The scale was adapted for the present study with permission from the first author. Respondents indicated the frequency of ICT-related activities (e.g., text messages, phone calls, surf the Internet, etc.) with response options ranging from (1) “never” to (5) “almost daily”. Scores for the 16 items were summed to create an overall score of ICT use, with higher scores indicating more use. Cronbach’s alpha reliability for the scale was 0.93.

#### 6.2.3. Time Spent on the Internet

Time spent on the Internet was assessed by three items. Respondents reported the time spent on the Internet on a normal university day, then the time spent on a day off (e.g., Sunday or holiday) and third, time spent each day on Social Networking Sites (e.g., Facebook, Twitter, etc.).

#### 6.2.4. Cyberbullying and Cyber Victimization Scales

The research on cyberbullying is in its infancy in Pakistan and no prior measure exists to assess cyberbullying in the Pakistani context. Therefore, the scales were developed for this study to investigate cyberbullying and victimization in university students. For item generation and design of the scales, we were guided by a previous study [56]. In addition, to explore the nature of cyberbullying and victimization in Pakistani university students, semistructured interviews were conducted with the 52 males and 42 females [57]. The analysis identified major themes, which were the basis for items on the scale. The final versions of The Cyberbullying and Cyber Victimization Scales consist of 20 Likert-type items for each scale. Response options range from 0 “Never” to 4 “More times a week.” Cronbach’s alpha reliability for the Cyberbullying and Cyber Victimization scale were 0.86 and 0.83, respectively.

## 7. Results

A CFA was conducted with the second sample using MPlus version 7.0 (Muthén & Muthén, 2012), with Maximum Likelihood (ML) estimator. The criteria to retain items on their respective factors were set at item loading ≥0.40. The results indicated a good model fit of the data with *χ*^2^ (*df*) = 1206.31 (132), *p* < 0.0005, Cumulative Fit Index (CFI) = 0.91, Tucker–Lewis Fit Index (TLI) = 0.90, standardized root mean squared residual (SRMR) = 0.04, and Root Mean Square Error of Approximation (RMSEA) = 0.08. Generally, values of CFI and TLI of 0.90 and above are considered to be an acceptable fit [58], 0.08 or below for RMSEA [59], and 0.08 or below for SRMR [58]. All items loaded above the criteria on the respective factors: for “Privacy and Security” loadings ranged from 0.68 to 0.81, for “Differentiation and Learning” loadings ranged from 0.66 to 0.77, and “Communication” loadings ranged from 0.48 to 0.74. Thus, the factor structure obtained in study 1 was confirmed in a larger independent sample. Internal consistency of the factors was tested using Cronbach’s alpha. The results presented in Table 1 show that the ICT Self-Efficacy Scale has excellent reliabilities ranging from 0.83 to 0.93, except for the Communication subscale (α = 0.67). The low reliability of that factor is likely because the scale consists of only three items.

To assess the convergent validity of the ICT Self-Efficacy Scale, Pearson bivariate correlations were computed with general self-efficacy. The results presented in Table 2 showed that general self-efficacy is moderately positively correlated with all three subscales including Privacy and Security (*r* = 0.43, *p* < 0.01) and Differentiation and Learning (*r* = 0.43, *p* < 0.01) and Communication (*r* = 0.27, *p* < 0.01). Additionally, the composite scale score of the ICT self-efficacy scale was moderately correlated (*r* = 0.50, *p* < 0.01) with general self-efficacy. These results support the convergent validity of the instrument in measuring ICT self-efficacy of the participants. The descriptive statistics of all study variables has been provided in Table 3.

Finally, relationships were explored between the ICT Self-Efficacy Scale, the time (frequency) variables, and Cyberbullying and Cyber victimization. Due to the skewed data in these variables, Spearman rho was calculated instead of the Pearson correlation coefficient. Results (see Table 4) show that the Privacy and Security subscale (*r* = 0.08, *p* < 0.01), and ICT self-efficacy total score are significantly positively correlated with time spent online on regular days (*r* = 0.18, *p* < 0.01), whereas time spent on an off day (e.g., weekend or holiday) is positively correlated with all scales of ICT self-efficacy and the composite score of ICT self-efficacy (*r* range 0.10 to 0.16, *p* < 0.01). Time spent on social networking sites was also positively correlated with “Privacy and Security” and “Communication” subscales along with the composite score of ICT self-efficacy (*r* = 0.09).

Moreover, a significant relationship was found between Cyberbullying, “Privacy and Security” (*r* = 0.09, *p* < 0.01), “Communication” (*r* = 0.13, *p* < 0.01), and the composite ICT self-efficacy score (*r* = 0.11, *p* < 0.01), but there was a nonsignificant relationship between Cyberbullying and “Differential and Learning subscale.” In addition, scores on cyber victimization were also significantly and positively associated with the “Communication” subscale (*r* = 0.14, *p* < 0.01), yet negatively (and non-significantly) related with “Privacy and Security” and “Differentiation and Learning”. Further, the relationship between cyber victimization and the composite score of ICT self-efficacy was nonsignificant.

## 8. Discussion

To our knowledge, this is the first empirical study related to cyberbullying and ICT self-efficacy with participants from a developing country. It is also one of a few quantitative studies to focus on cyberbullying in a university context. These factors, along with the rigorous step-by-step development of the measure of ICT self-efficacy, make a unique contribution to the literature.

The current research has contributed to the development and validation of an ICT self-efficacy scale. Following Bandura’s [19] recommendation, this situates ICT self-efficacy in the context of cyberbullying and victimization. The three-factor solution emerging in the first study and confirmed in the second study provides evidence for multidimensionality of ICT self-efficacy. Content analysis of the first factor (i.e., Privacy and Security) clearly shows that the factor addresses both basic and advanced skills for perceiving control in cyberspace. Items constituting the second factor (i.e., differentiation and learning) indicate the individual’s evaluating capacity, and their learning ability to further gain control of the cyber world they inhabit. Finally, the third factor consisted of items indicating the individual’s ability and behavior of both verbal and visual communication in the cyber world. Findings provided evidence of content validity, internal consistency, and factorial reliability of the scale. Moreover, the scores on the ICT self-efficacy scale were positively related to the measure of general self-efficacy, time spent online on weekdays, time spent on off days (i.e., weekends or holidays) and time spent on SNS. Consequently, the scale appears to be a useful and valid three-factor measure to assess ICT self-efficacy in the context of cyberbullying.

Using this scale of ICT self-efficacy that is tailored for the cyber context, we found that a strong association exists between perpetration of cyberbullying and the composite score on ICT self-efficacy. This is consistent with existing studies that have shown an association of cyberbullying and greater online expertise [13], and computer skills [15]. This is also consistent with Savage and Tokunaga [28], who found no significant association of perpetration of cyberbullying when ICT self-efficacy was low. In addition, these findings provided evidence that the association between self-efficacy and bullying is not limited to traditional bullying [60]; the domain-specific, i.e., ICT self-efficacy, has unique contributions with relevance to cyberbullying.

Second, a significant relationship was found between perpetration of cyberbullying and the subscale “Privacy and Security” on the ICT self-efficacy scale. This demonstrates that cyberbullies possess greater confidence and capability to use privacy and security features. It may be that once engaging in cyberbullying, they anticipate retaliation in response and, as a precautionary measure, have greater knowledge and confidence in using privacy and security features of ICT.

Third, both cyberbullying and cyber victimization were found to be associated with the “Communication” subscale. This finding is expected because most cyberbullying and victimization occurs on communication platforms. Students mostly use social platforms and media tools for interaction and communication purposes [61]. Fourth, cyber victimization was found to be negatively but nonsignificantly associated with “Privacy and Security.” Though the relationship was nonsignificant, the negative relationship suggests that cyber victims may have less confidence in their ability to use privacy and security features of ICT. This is consistent with the absence of a relationship between cyber victimization and the composite score of ICT self-efficacy.

The findings of this study are crucial for designing and enhancing intervention efforts. The current research is an initial step to highlight the significance of ICT self-efficacy in cyberbullying research. In a broader context, ICT self-efficacy should be considered relevant for the prevention of cyberbullying and victimization. Few anticyberbullying programs [62,63] have shown success in reducing cyberbullying and victimization, and none have focused on ICT self-efficacy. Prevention programs might incorporate hands-on practice as well as demonstrations to enhance ICT self-efficacy with a special focus on teaching online safety and security-related skills. It seems that those inclined to cyberbully others acquire those skills, but those who may be targeted would benefit from specific instruction on those skills.

### 8.1. Limitations and Future Directions

The primary limitation of the present research is its cross-sectional design. A longitudinal study would provide evidence for reciprocal causality and greater predictive ability. Thus, future research should include multiple waves of data over sufficient time to better understand this relationship. Second, although the alpha reliability for the “Communication” dimension is (α = 0.67) within the range of minimally acceptable reliability criteria [64], it is slightly lower than the revised criteria provided by Nunnally [45]. Theoretically, it has been argued that the larger the number of items in a scale, the more reliable will be the scale [45]. Therefore, in order to increase the reliability, future studies may generate and evaluate more items to fully cover the domain of Communication ICT self-efficacy. Third, establishing the validity of a construct is continuous and ongoing process. The present study only utilized samples of Pakistani university students, while the measure could be validated on cross-national samples and on different age groups. Fourth, it is important that future research should include traditional bullying as a control variable per the recommendation of Olweus [7], and then investigate which type of self-efficacy contributes more to cyberbullying and victimization: general, or ICT domain-specific. It would also be interesting to know whether general self-efficacy is more associated with traditional bullying or cyberbullying. Finally, investigating potential moderators and mediators of the relationship between ICT self-efficacy and cyberbullying and cyber victimization would extend our understanding of these phenomena.

Future research on this measure should include analyses of factorial invariance across genders and cross-national studies with both other developing countries and developed countries; invariance of the measure across cultures should also be tested.

### 8.2. Conclusions

The present study contributed to the cyberbullying literature by developing and testing a rigorous measure of ICT self-efficacy and providing evidence of its content, factorial and convergent validity, as well as its association with cyberbullying and victimization. The study lays the foundation for further research to understand the comprehensive role of domain-specific ICT self-efficacy concerning cyberbullying.

## Figures and Tables

**Table 1:** Factor analysis of ICT Self-Efficacy Scale.

S. No.	Item No.	Statements	Factor Loadings	
		Factors	EFA	CFA
		**Factor 1: Privacy and Security (α = 0.89; 0.93)**		
1	15	I can easily hide any post that someone shared/tagged on my profile on social networking sites that I mostly use (i.e., Facebook, etc.)	0.87	0.80
2	13	I can easily block or restrict anyone on social networking sites that I mostly use (i.e., Facebook, twitter, Skype, WhatsApp, Viber etc.)	0.83	0.79
3	17	I can easily set pins/password on my mobile phone to keep it secure.	0.81	0.75
4	18	I can easily change password of my email/social networking account that I mostly use.	0.81	0.81
5	14	I can easily unfriend anyone on social networking sites that I mostly use (i.e., Facebook, Twitter, Skype, WhatsApp, Viber, etc.)	0.79	0.81
6	16	I can easily report a fake account pretending to be me.	0.76	0.70
7	12	I can easily report any ID, post, image or video as abusive/spam content on social networking sites that I mostly use (i.e., Facebook, Twitter, Skype, WhatsApp, Viber, etc.)	0.75	0.76
8	11	I can easily control privacy settings of social networking sites that I mostly use (i.e., Facebook, Twitter, Skype, WhatsApp, Viber, etc.)	0.71	0.76
9	19	I can easily recover my email/social networking account if I forget the password.	0.58	0.72
10	20	I can easily handle spams that I received through email or posted on my wall on social networking site (i.e., Facebook, etc.)	0.54	0.68
		**Factor 2: Differentiation and Learning (α = 0.81; 0.83)**		
11	4	I can easily judge whether the information that someone has provided on social networking sites is correct.	0.87	0.66
12	3	I can easily judge trustworthy information on social networking sites (i.e., Facebook, Twitter, etc.)	0.85	0.73
13	5	I am fully aware of the consequences of my conduct on the Internet.	0.71	0.68
14	1	I can easily express my point of view on any online discussion forum.	0.65	0.67
15	2	When I open any website, I can easily learn in a very short time how to use its features/functions.	0.64	0.77
		**Factor 3: Communication (α = 0.67; 0.67)**		
16	9	I can easily use chat rooms on the Internet.	0.84	0.69
17	7	I can easily talk to others through the Internet using a webcam.	0.74	0.48
18	8	I can easily edit or modify any picture on the computer/mobile phone using different software (i.e., Photoshop, etc.)	0.55	0.74
		Cronbach Alpha for the composite scale (α = 0.93; 0.92)		

EFA: exploratory factor analysis; CFA, confirmatory factor analysis; Cronbach’s alphas are reported in parentheses for Study 1 and Study 2, respectively.

**Table 2:** Pearson bivariate correlations between ICT Self-Efficacy and General Self-Efficacy.

Variables	1	2	3	4	5
1. Privacy & Security	-	0.61 **	0.43 **	0.94 **	0.44 **
2. Differentiation & Learning		-	0.43 **	0.80 **	0.49 **
3. Communication			-	0.63 **	0.27 **
4. ICT Self-Efficacy				-	0.50 **
5. General Self-Efficacy					-

** *p* < 0.01.

**Table 3:** Descriptive statistics of the study variables.

Variables		Range					
	Items	Potential	Actual	*M*	*SD*	Skew	Kurt
Privacy & Security	10	10–50	10–50	31.10	9.41	−0.31	−0.41
Differentiation & Learning	5	5–25	5–25	16.63	4.35	−0.53	−0.01
Communication	3	3–15	3–15	8.77	2.96	−0.07	−0.62
ICT Self-Efficacy	18	18–90	18–88	60.77	14.52	−0.86	0.44
General Self-Efficacy	10	10–40	10–40	28.39	6.35	−0.49	0.07
Social Desirability	16	0–16	0–16	11.19	2.87	−0.57	0.20
Time Spent Online (Weekdays)	-	-	0.03–10	2.46	2.03	1.52	2.01
Time Spent Online (off days)	-	-	0.08–11.8	5.02	3.10	0.71	−0.41
Time Spent on SNS	-	-	0.08–10.95	2.73	2.28	1.56	2.15
Cyberbullying	20	0–80	0–37.89	4.04	6.27	2.31	6.15
Cyber Victimization	20	0–80	0–64.21	8.87	7.84	1.80	4.93

**Table 4:** Spearman Rho correlation between ICT Self-Efficacy, time variables, and Cyberbullying and Cyber Victimization.

Variables	1	2	3	4	5	6	7	8	9
1. Privacy and Security	-	0.54 **	0.40 **	0.92 **	0.10 **	0.15 **	0.08 **	0.09 **	−0.02
2. Differentiation and Learning		-	0.38 **	0.74 **	0.06	0.10 **	0.02	0.01	−0.03
3. Communication			-	0.62 **	0.01	0.14 **	0.08 **	0.13 **	0.14 **
4. ICT Self-Efficacy				-	0.08 **	0.18 **	0.09 **	0.11 **	0.03
5. Time Spent Online (Weekdays)					-	0.45 **	0.37 **	0.20 **	0.16 **
6. Time Spent Online (off days)						-	0.49 **	0.27 **	0.28 **
7. Time Spent on SNS							-	0.24 **	0.33 **
8. Cyberbullying								-	0.58 **
9. Cyber Victimization									-

** *p* < 0.01.
